# PLC and PAD2 Regulate Extracellular Calcium‐Triggered Release of Macrophage Extracellular DNA Traps

**DOI:** 10.1002/eji.202350942

**Published:** 2025-04-01

**Authors:** Neha Mishra, Magdalena Mohs, Nico Wittmann, Stefan Gross, Paul R. Thompson, Lukas Bossaller

**Affiliations:** ^1^ Section of Rheumatology, Department of Medicine A University Medicine Greifswald Greifswald Germany; ^2^ Section of Pediatric Rheumatology, Department of Pediatric and Adolescent Medicine University Medicine Greifswald Greifswald Germany; ^3^ Department of Internal Medicine B University Medicine Greifswald Greifswald Germany; ^4^ Department of Biochemistry and Molecular Biotechnology University of Massachusetts Medical School Worcester USA

**Keywords:** cell death, citrullination, extracellular calcium, inflammasomes, macrophage extracellular DNA traps

## Abstract

Macrophages can respond to infection or cellular stress by forming inflammasomes or by releasing extracellular traps (ETs) of DNA through METosis. While ETs have been extensively studied in neutrophils, there are fewer studies on METosis. We show that extracellular calcium and LPS enable human monocyte‐derived macrophages (hMDM) to release extracellular DNA decorated with myeloperoxidase (MPO) and citrullinated histone, alongside ASC aggregation and IL‐1ß maturation, indicating NLRP3 inflammasome activation. Compared with m‐CSF differentiated macrophages only gm‐CSF differentiated macrophages expressed macrophage elastase (MMP12) and METs released by the latter had significantly more bactericidal activity toward *E. coli*. Mechanistically, phospholipase C and peptidyl arginine deiminase‐2 inhibition attenuate MET release. Interestingly, NLRP3 inflammasome blockade by MCC950 had a significant effect on MET release. Finally, MET release was completely blocked by plasma membrane stabilization by punicalagin. Altogether, we demonstrate that extracellular calcium‐activated hMDM extrude DNA, containing citrullinated histones, MPO, MMP12, and ASC specks and released METs kill bacteria independent of hMDM phagocytotic activity. We believe that calcium‐activated hMDM adds a physiologically relevant condition to calcium ionophore induced cell death that may be important in autoimmunity.

## Graphical abstract

Extracellular calcium induces MET release from human macrophages. Concurrently, extracellular calcium activates the NLRP3 inflammasome. Citrullinated histones, MMP12, MPO and ASC specks are bound to METs and are released by rupture of the plasma membrane, where they exhibit bactericidal properties.

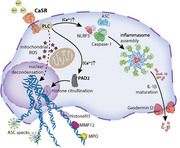

**Abbreviations**
ASC: apoptosis‐associated speck‐like protein containing a caspase recruitment domain
gm‐CSF: granulocyte‐macrophage colony‐stimulating factor
GSDMD: gasdermin D
hMDM: human monocyte‐derived macrophage
LPS: lipopolysaccharide
m‐CSF: macrophage colony‐stimulating factor
METs: macrophage extracellular traps
mito‐ROS: mitochondrial reactive oxygen species
MMP12: matrix metalloproteinase‐12 or macrophage elastase
MPO: myeloperoxidase
NETs: neutrophil extracellular traps
NLRP3: nucleotide‐binding domain leucine‐rich repeat‐containing receptor 3
PAD2: peptidylarginine deiminase 2
PLC: phospholipase C
ROS: reactive oxygen species

## 1 Introduction

Web‐like structures composed of extracellular nuclear or mitochondrial DNA ejected from immune cells are called ETs. These play an important role in host immune defense during infection and inflammation and were first described in neutrophils [1]. Neutrophils can capture and kill microbes through the release of neutrophil extracellular traps (NETs) in addition to their phagocytic capacity and degranulation activity [1]. NETs essentially constitute of broken‐down nuclear content attached with histones, enzymes, for example, myeloperoxidase (MPO) and neutrophil elastase (NE), proteins, for example, lactoferrin or cathepsins [1] and many other cytoskeletal and cytoplasmic proteins [2, 3]. Mechanistically several models for NET generation following neutrophil activation have been described. Clearly an NADPH oxidase‐dependent ROS production pathway exists, which promotes NE and MPO translocation from “azurosome” to the nucleus. In the nucleus, NE and MPO bind to chromatin and cleave histones promoting chromatin decondensation [4]. However, depending on the stimuli studied, NADPH oxidase‐independent NETosis occurs in the absence of NADPH oxidase or MPO activity [5, 6, 7]. Histones are citrullinated during NETosis which further promotes chromatin decondensation in a peptidyl arginine deiminase 4 (PAD4) dependent process [5, 8]. Although histones are citrullinated, the role of PAD enzymes is not very well established since histone citrullination may not be essential for NET release [6]. In the later stages of NETosis, release of DNA into the extracellular space and neutrophilic lytic cell death occurs through NE‐dependent gasdermin D (GSDMD) activation which form pores in the nuclear and plasma membranes [9]; however, the requirement for GSDMD pores in the process of NETosis has recently been challenged [10, 11] and further complicating matters, NETosis can also progress in a nonlytic form depending on the stimuli employed [12, 13, 14, 15].

Cleaved GSDMD also induces plasma membrane pores to promote inflammatory pyroptotic cell death in macrophages [16, 17]. Pyroptosis is triggered by a signaling platform known as inflammasomes which are assembled in response to the recognition of pathogen‐associated molecular patterns or self‐derived danger‐associated molecules (DAMP). These large multiprotein complexes oligomerize with sensors such as NLRP1, NLRP3, NLRC4, AIM2, and the adaptor protein ASC to recruit inflammatory caspase‐1 and induce its autoproteolytic cleavage. Mature caspase‐1, in turn, cleaves and activates GSDMD and proinflammatory IL‐1ß and IL‐18 cytokines, which are released into the extracellular space through GSDMD pores [16, 17, 18].

Similar to neutrophils, an antimicrobial function of extracellular DNA released from mast cells [19] and from eosinophils [20] has been reported. Moreover, monocytes are capable of releasing extracellular DNA in response to microbial stimuli [21]. Macrophages can also produce extracellular traps (METs) following muscle necrosis [22] and during infections [23, 24], and the role of METs in inflammatory diseases has been reviewed recently by Rasmussen and Hawkins [25]. Interestingly, extracellular calcium has been suggested to play a role in METosis secondary to *Mycobacterium massiliense*‐infected human THP‐1 cells [26] and extracellular calcium is also well known to trigger NLRP3 inflammasome activation in macrophages [27, 28, 29]. However, little data exist on the role of PAD enzyme‐dependent histone citrullination and MET release in macrophages, especially using physiologically relevant concentrations of extracellular calcium. PAD2 inhibition was shown to reduce MET release and reduce liver metastasis [30]. In contrast, MET release in response to hypochlorous acid from human macrophages was shown to be independent of PAD2 using a siRNA knockdown approach [31] whereas another study found a dependency on PAD4 in THP‐1 differentiated macrophages [32].

Overall, in comparison to neutrophils, the cues that trigger extracellular DNA release in macrophages have received limited attention and their essential characteristics have not been clearly defined in humans. In this study, we therefore investigated the capacity of human monocyte‐derived macrophages (hMDM) to release extracellular DNA in response to extracellular calcium and lipopolysaccharide (LPS). We show that following a pyroptotic stimulus with extracellular calcium, macrophages release extracellular traps with strands composed of chromosomal DNA, citrullinated histones, MMP12, MPO, and extracellular ASC specks. Similar to NETs, METs possess antibacterial properties.

## 2 Results

### 2.1 Macrophages Release Extracellular Traps in Response to Extracellular Calcium

**FIGURE 1:**
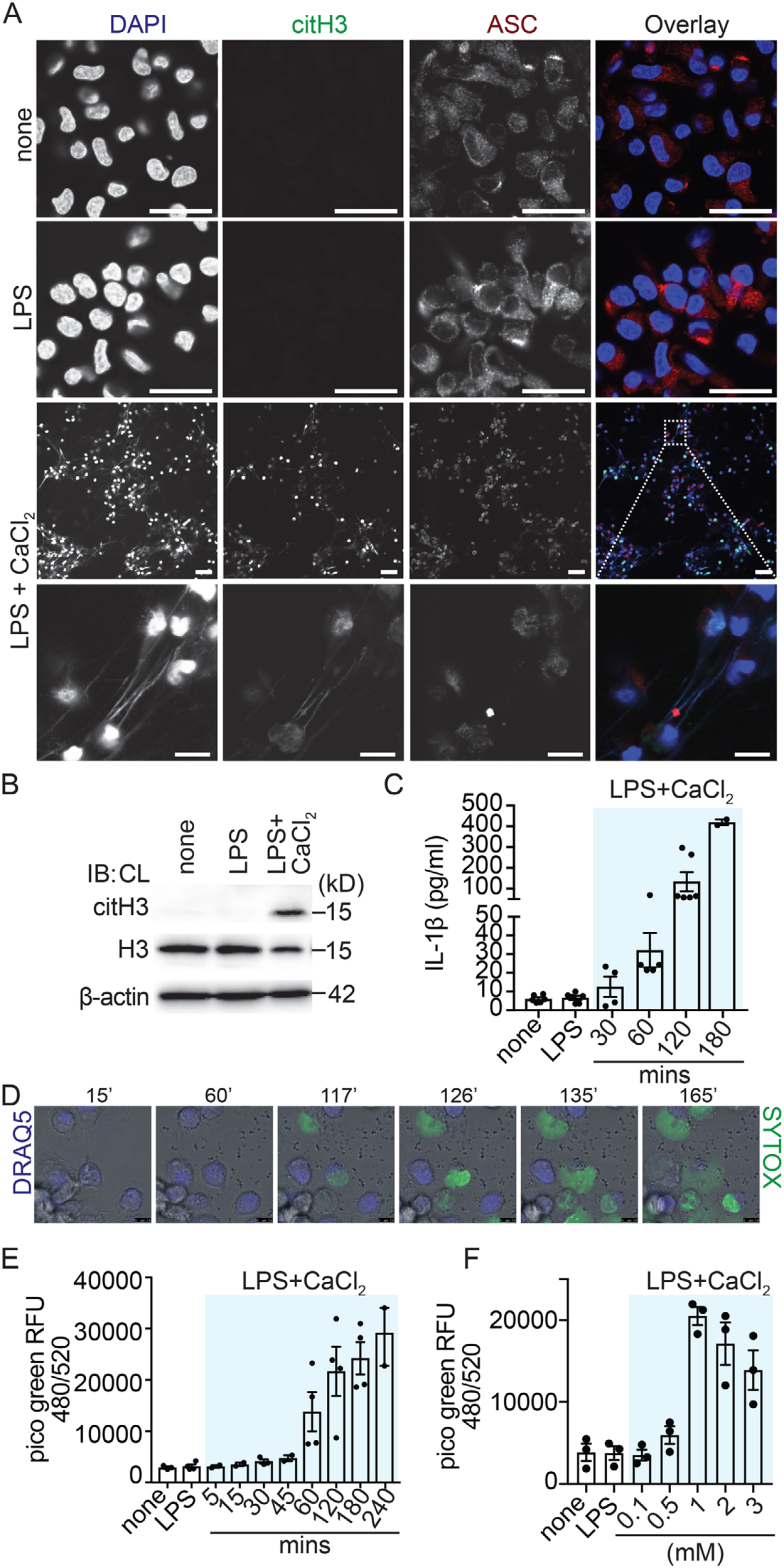
Extracellular calcium induces inflammasome activation together with MET release: (A) Confocal microscopy of gm‐CSF macrophages stained for DNA (blue), citH3 (green), and ASC (red) under resting conditions or LPS‐primed (1 ng/mL) for 2 h or LPS primed and stimulated for 3 h with extracellular Ca^2+^ (1 mM) (scale bar all 25 µM), 10× magnification of the outlined area at the right displayed in the bottom row images (scale bar 10 µM). (B) Immunoblot for citH3 and histone H3 from cell lysates of macrophages treated with the indicated conditions; ß‐actin was used as a loading control. (C) ELISA for IL‐1ß of supernatants from inactivated (none), LPS primed (1 ng/mL) and extracellular Ca^2+^ (1 mM) activated hMDM for 30 to 180 min as indicated. (D) Live cell imaging of gm‐CSF hMDM stained with DRAQ5 (blue) and Sytox Green (green) primed with LPS (1 ng/mL) and activated for 3 h with extracellular Ca^2+^ (1 mM). (E) Quantification of METs from inactivated (none) or from LPS‐primed or from LPS‐primed and extracellular Ca^2+^ (1 mM) activated gm‐CSF macrophages for 5 to 240 min as indicated. (F) Quantification of METs stimulated for 3 h with 0.1 to 3 mM extracellular Ca^2+^ as indicated. Data information: Each dot represents an individual biological replicate (C, E, F). Data are representative of five independent experiments (A) or representative of one out of four independent experiments (band densitometry part of Figure 5G) (B) or representative of three or four independent experiments (C) or representative of one from three independent experiments (D) or two independent experiments with two technical replicates (E) or three independent experiments with three technical replicates (F). Data are presented as the mean and SEM.

We have previously reported that protein citrullination is common during pyroptotic cell death in murine macrophages [33]. Therefore, we were interested if histone citrullination, a marker of extracellular trap release in neutrophils, would also occur during pyroptotic cell death. Since protein citrullination is dependent on Ca^2+^‐activated PAD enzymes and extracellular Ca^2+^ was required for citrullination in our previous study, we stimulated hMDM with LPS or LPS with extracellular Ca^2+^. As expected, neither in the unstimulated nor in LPS‐primed human macrophages, histone H3 citrullination was observed via confocal microscopy and immunoblotting (Figure 1A,B). This result contrasts with extracellular trap release from neutrophils, where LPS alone is already sufficient to cause histone citrullination in neutrophils [34]. However, surprisingly we detected prominent histone H3 citrullination in human macrophages primed for 2 h with 1 ng/ml LPS and exposed to 1 mM extracellular Ca^2+^ for 3 h (Figure 1A,B), a condition that also induces pyroptotic cell death via stimulation of the calcium‐sensing receptor (CASR; [27, 33]). Pyroptotic cell death was observed via ASC redistribution by confocal microscopy, which is homogenous cytoplasmic in unstimulated and LPS‐primed macrophages, into ASC specks in LPS and extracellular Ca^2+^ activated macrophages (Figure 1A), via IL‐1ß release into the supernatant by ELISA (Figure 1C) and GSDMD cleavage (Figure S1A). Surprisingly, the release of extracellular DNA decorated with citrullinated histone was observed in some ASC speck‐positive hMDM, indicating inflammasome activation alongside MET formation in these cells. Strikingly, we found ASC specks associated with extracellular DNA in some cases (Figure 1A, inset, Video S1). On a single‐cell level via live time‐lapse confocal microscopy, hMDM displayed decondensation of nuclear chromatin before MET formation (Figure 1D, Video S2).

Next, we were interested in further quantifying the release of extracellular DNA after stimulation with extracellular Ca^2+^. Using Picogreen to quantify extracellular DNA release after DNase digestion [35], we found that macrophages released DNA from roughly 60 min onward and DNA release further increased for up to 4 h (Figure 1E). Importantly, we did not detect a significant release of extracellular DNA when hMDM were stimulated with LPS alone (Figure S1B). hMDM stimulated with LPS and nigericin released some extracellular DNA compared with LPS only (Figure S1C); however, the difference in the magnitude of extracellular DNA release comparing the LPS and nigericin condition to the LPS and extracellular Ca^2+^ condition was significantly higher (Figure S1C), underscoring the key role extracellular calcium has in this process.

To identify the optimal extracellular Ca^2+^ concentration for MET release, hMDM were exposed to increasing extracellular Ca^2+^ concentrations. Already low extracellular Ca^2+^ concentrations resulted in minimal DNA release from macrophages; however, DNA release peaked at 1 mM Ca^2+^ and declined thereafter (Figure 1F). We also examined, if MET release can be triggered in the absence of LPS via extracellular Ca^2+^ only (Figure S1D). Under this condition, we observed extracellular DNA release comparable to stimulation with LPS and extracellular Ca^2+^, suggesting that MET formation is independent of LPS priming.

Together, these data establish that extracellular Ca^2+^ can trigger extracellular DNA release in hMDM.

### 2.2 METs Are Composed of Myeloperoxidase and MMP12

**FIGURE 2:**
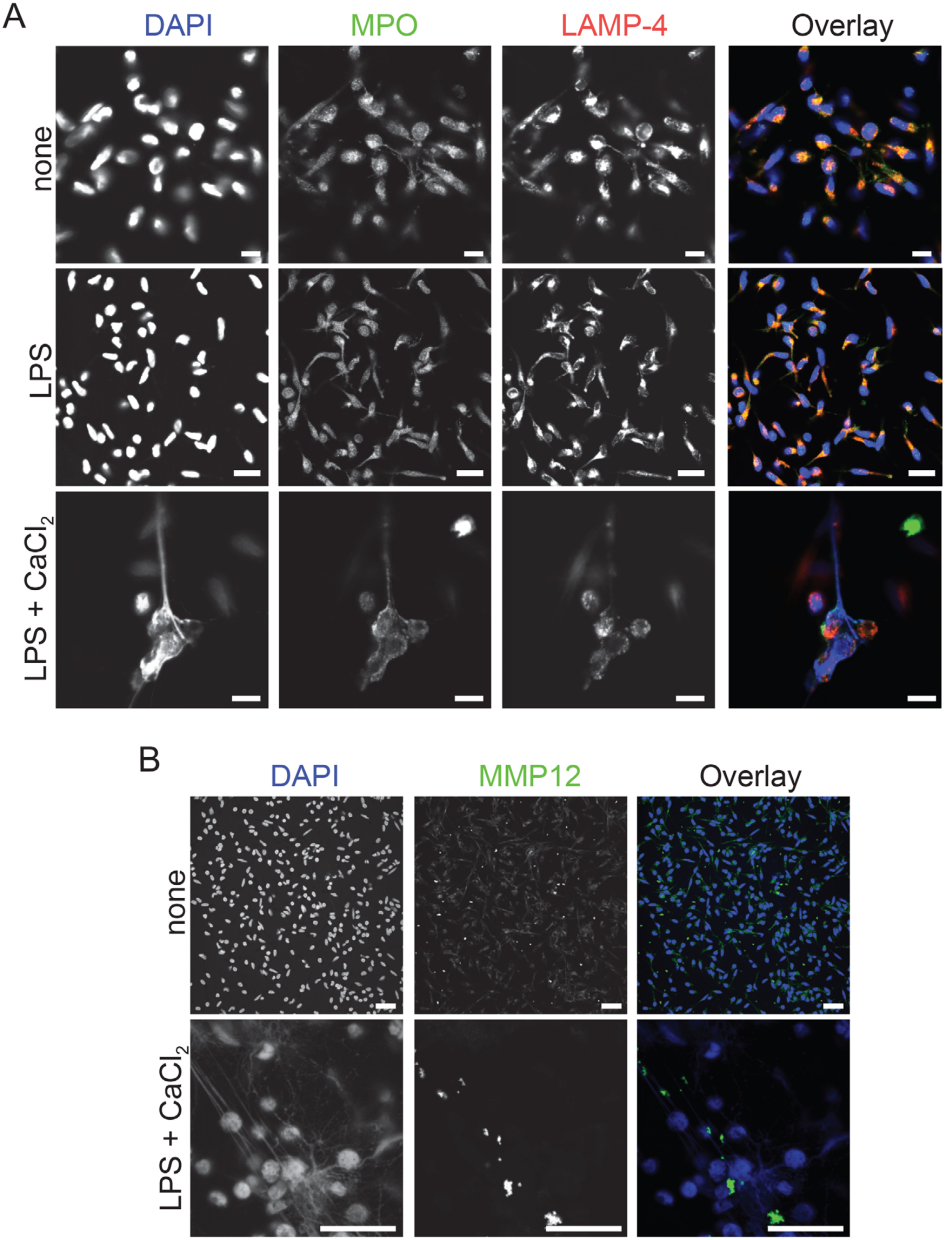
Myeloperoxidase and matrix metalloproteinase‐12 are associated with METs: (A) Confocal microscopy of gm‐CSF‐hMDM stained for DNA (blue), MPO (green), and LAMP‐4 (red) and either left untreated (none) (scale bar 5 µM) or primed for 2 h with LPS (1 ng/mL) (scale bar, 7.5 µM) or primed and stimulated for 3 h with extracellular Ca^2+^ (1 mM) (scale bar 5 µM) or (B) stained for DNA (blue) and MMP12 (green) and DNA (blue) from inactivated (none) (scale bar 25 µM) or LPS primed and extracellular Ca^2+^ activated hMDM (scale bar 25 µM). Data information: Data are representative of three independent experiments (A) or two independent experiments (B). Further confocal images availabe (supporting information “responses to the reviewers”).

Neutrophil‐derived NETs are composed of enzymes from azurophilic granules such as MPO, NE, hydrolases such as cathepsin G, and other bactericidal proteins [1]. Furthermore, NE and MPO enhance NET formation through partial histone degradation and chromatin decondensation [4]. Because under our assay conditions, citrullinated histones were associated with extracellular DNA extruded from macrophages (Figure 1A; Figure S2A; line analysis via pixel intensity [*y*‐axis] along the indicated line [*x*‐axis] for DAPI and citrullinated histone in Figure S2A and Sytox Green and DRAQ5 in Figure S2B), we were interested to examine whether MPO and macrophage elastase, MMP12, were also associated with METs in hMDM. The composition of METs was analyzed by confocal microscopy. In resting and LPS primed hMDM MPO colocalized with the endosomal marker LAMP‐4. Interestingly, in LPS primed and extracellular Ca^2+^ stimulated hMDM MPO was dissociated from LAMP‐4, suggesting the release of MPO into the cytosol. METs were also covered by MPO (Figure 2A; Figure S2C; line analysis) as well as MMP12 (Figure 2B; Figure S2D; line analysis). These results suggest that METs are composed of citrullinated histone, MPO and MMP12, and that MPO is released into the cytosol during METosis.

### 2.3 MPO Inhibition in gm‐CSF hMDM Leads to a Reduced Capacity to Release METs

**FIGURE 3:**
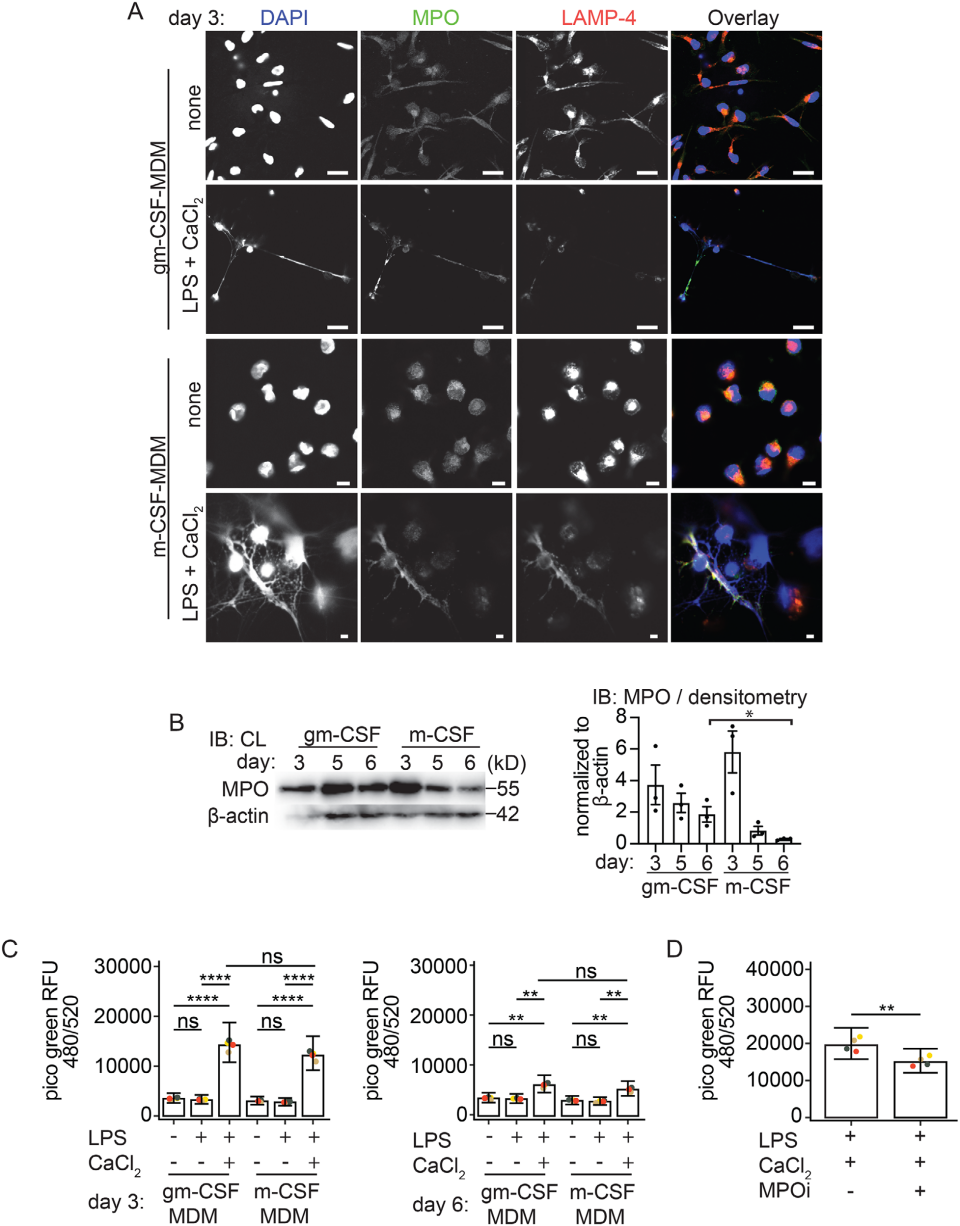
MPO inhibition in gm‐CSF hMDM leads to a reduced capacity to release METs: (A) Confocal microscopy of gm‐CSF or m‐CSF differentiated hMDM stained for MPO (green), LAMP‐4 (red) and DNA (blue) left untreated or LPS primed and stimulated for 3 h with extracellular Ca^2+^ (1 mM). (B) Immunoblot and quantification by band densitometry for MPO from cell lysates of gm‐CSF and m‐CSF hMDM differentiated for 3, 5, and 6 days. ß‐actin serves as a loading control. (C) Quantification of METs from gm‐CSF or m‐CSF hMDM differentiated for 3 or 6 days left inactivated (−) or from LPS‐primed (1 ng/mL) or LPS‐primed and activated with extracellular Ca^2+^ (1 mM). (D) Quantification of METs from 3 day gm‐CSF differentiated hMDM primed with LPS (1 ng/mL) and activated with extracellular Ca^2+^ (1 mM) or primed with LPS (1 ng/mL) and pretreated for 30 min with MPO inhibitor and stimulated for 3 h with extracellular Ca^2+^ (1 mM). Data information: Each dot represents an individual biological replicate (B–D). Data are representative of one from three independent experiments (A), data are representative of one from three independent experiment (B), data are representative of three independent experiments with technical triplicates (day 3) or from four independent experiments with technical triplicates (day 6) (C), data are representative of four independent experiments with technical quadruplicates (D). Data are presented as the mean and SEM (B), data presented as the mean and 95% confidence interval (C, D). ns, not significant, ‘****’ *p* < 0.0001, ‘***’ *p* < 0.001, ‘**’ *p* < 0.01, ‘*’ *p* < 0.05. (Generalized linear mixed models for Gamma‐family with log‐link function and Tukey‐adjusted post hoc test (C) or paired *t*‐test (D)). Scale bar, 10 µM.

Macrophages can acquire different cellular functions depending on the growth factors they are differentiated or polarized with. Gm‐CSF polarized hMDM are considered to be proinflammatory, while m‐CSF polarized hMDM are generally ascribed an anti‐inflammatory function. Therefore, we next investigated whether macrophage polarization has an impact on the ability to release METs. The surface expression of CD40, CD80, CD86, CD163, and CD206 for gm‐CSF and m‐CSF differentiated macrophages was confirmed via FACS analysis ([36]; Figure S3A) and was analyzed by confocal microcopy for expression of MPO, the macrophage marker LAMP‐4/CD68, citH3 and ASC. On day 3 of differentiation, unstimulated gm‐CSF‐hMDM and m‐CSF‐hMDM stained positive for LAMP‐4/CD68 and MPO (Figure 3A). LPS primed and extracellular Ca^2+^ activated gm‐CSF‐hMDM and m‐CSF‐hMDM released METs positive for MPO (Figure 3A). As expected, ASC redistribution to ASC specks (Figure S3B) and caspase‐1 cleavage in the supernatant in gm‐CSF‐MDM (Figure 5G) and m‐CSF‐MDM confirmed inflammasome activation (Figure S3C). Importantly, as with gm‐CSF differentiated MDM in the previous result section (Figure S1B) we again did not detect a significant release of extracellular DNA from m‐CSF differentiated hMDM stimulated with LPS alone (Figure S3D). LPS and nigericin‐stimulated m‐CSF differentiated MDM also showed a small increase in the release of extracellular DNA; however, the magnitude of the release was again much lower compared with the LPS primed and extracellular Ca^2+^ condition (Figure S3E). Therefore, MET release in response to LPS and extracellular Ca^2+^ is independent of the macrophage polarization state on day 3 differentiated macrophages.

It has previously been reported that gm‐CSF regulates MPO expression during macrophage differentiation [37]. Sugiyama et al. showed that MPO expression decreases in monocyte‐derived m‐CSF polarized macrophages during prolonged differentiation beyond day 3. Importantly, MPO is known to promote extracellular DNA release in neutrophils [4]. We were therefore interested in investigating whether a decline in MPO expression in a 6‐day culture may influence the ability of m‐CSF‐differentiated hMDM to release METs and could thereby give some mechanistic hints at how METs are regulated in human macrophages. First, we investigated whether the expression of MPO in gm‐CSF‐hMDM differs from that in m‐CSF‐hMDM when they are compared during differentiation for 3 to 6 days. As reported by Sugiyama et al., we also observed a decrease in MPO expression over time via immunoblotting; however, MPO was not completely lost (Figure 3B).

Given that on day 6 m‐CSF‐differentiated hMDM had reduced levels of MPO compared to preserved MPO levels at day 3, we then studied MET induction by Picogreen assay in LPS primed and extracellular calcium stimulated hMDM. We did not observe a significant reduction in extracellular DNA extruded from day 6 m‐CSF‐hMDM compared with day 6 gm‐CSF differentiated hMDM (Figure 3C). However, we observed that in the presence of the MPO inhibitor, the release of extracellular DNA was significantly reduced in the gm‐CSF hMDM (Figure 3D), whereas no inhibition of extracellular DNA release was observed in the calcium‐activated gm‐CSF hMDM (data not shown). Therefore, our data suggest that extracellular DNA release is partially MPO‐dependent in LPS‐primed and extracellular calcium‐activated gm‐CSF hMDM.

### 2.4 The Antibacterial Protein MMP12 Is Expressed in Gm‐CSF hMDM and Gm‐CSF‐derived METs Have a Significantly Enhanced Killing Capacity Toward Extracellular Bacteria

**FIGURE 4:**
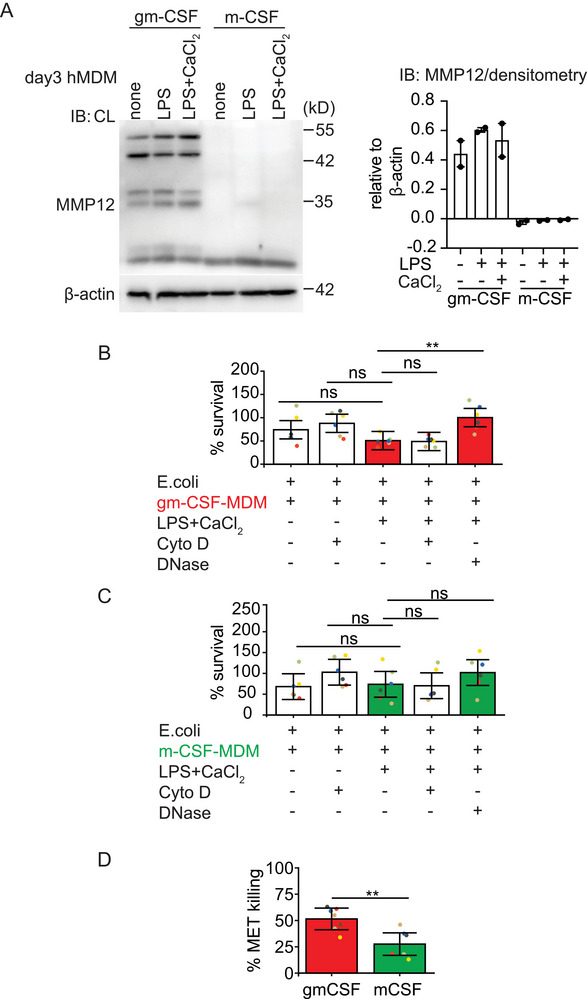
MMP12 is expressed in gm‐CSF hMDM and gm‐CSF hMDM derived METs have higher antibacterial properties: (A) Immunoblot and quantification by band densitometry for MMP12 from cell lysates of untreated or LPS‐primed (1 ng/mL) or LPS‐primed and extracellular Ca^2+^ (1 mM) stimulated gm‐CSF and m‐CSF hMDM differentiated for 3 days. (B) % survival of *E. coli* incubated with gm‐CSF hMDM or (C) m‐CSF hMDM left untreated or treated with cytochalasin D or stimulated with LPS plus extracellular Ca^2+^ or stimulated with LPS plus extracellular Ca^2+^ and treated with cytochalasin D or stimulated with LPS+ extracellular Ca^2+^ and treated with DNase. (D) % of *E. coli* killing in gm‐CSF and m‐CSF hMDM in LPS primed and activated with extracellular Ca^2+^. Data information: Each dot represents an individual biological replicate (B–D). Data are representative of one out of two independent experiments (A), of six independent experiments with two or three technical replicates (B, C). Data are presented as the mean and 95% confidence limits. ns, not significant, ‘****’ *p* < 0.0001, ‘***’ *p* < 0.001, ‘**’ *p* < 0.01, ‘*’ *p* < 0.05. Linear mixed models with Tukey‐adjusted post hoc test (B, C) or paired *t*‐test (D).

Macrophages are known for their ability to phagocytose and kill pathogens. MMP12 is one such proteinase that contributes to antibacterial properties in macrophages [38]. Intracellular stores of MMP12 are mobilized after bacterial uptake to macrophage phagolysosomes where it disrupts the bacterial cell membrane, resulting in bacterial death. MMP12 is also secreted as a proenzyme and subsequently activated in the extracellular space to perform its biological function [38]. To investigate whether MMP12 protein levels are differentially regulated by gm‐CSF or m‐CSF, we compared gm‐CSF with m‐CSF‐differentiated hMDM on day 3 for MMP12 expression by immunoblot, a timepoint in differentiation in which both types of macrophages still express equal amounts of MPO. In line with data by Aristorena et al. [39] and Fuentelsaz‐Romero et al. [40], m‐CSF hMDM were deficient in MMP12 protein expression (condition “none” in Figure 4A]. MMP12 was also absent in m‐CSF hMDM under MET‐inducing conditions with LPS and extracellular calcium (Figure 4A). MMP12 deficiency had no influence on MET release, as reported in the previous result section (Figure 3C).

We then examined the ability of METs from day 3 differentiated macrophages to kill *E. coli*, thereby comparing MMP12^+^MPO^+^ gm‐CSF hMDM to MMP12^‐^MPO^+^ m‐CSF hMDM. Gm‐CSF‐ and m‐CSF‐differentiated hMDM were activated with LPS and extracellular calcium to induce MET release. As a control condition and to distinguish the bactericidal activity of METs from the phagocytotic ability of macrophages, phagocytosis was blocked by the addition of cytochalasin D and *E. coli* were added thereafter for 1 h to unstimulated or LPS and extracellular calcium stimulated hMDM. Further, to prove that extracellular DNA had bactericidal activity, recombinant DNase 1, an endonuclease digesting extracellular DNA, was added for 15 mins into the culture media to degrade METs prior to the addition of *E. coli*. Bacterial survival was significantly decreased with METs produced by MMP12^+^MPO^+^ gm‐CSF hMDM and was restored in the presence of DNase 1 (Figure 4B). On the contrary, METs from MMP12^‐^MPO^+^ m‐CSF hMDM did not significantly decrease bacterial survival (Figure 4C). When we compared the percent survival of *E. coli* from LPS and Ca^2+^ activated gm‐CSF versus m‐CSF MDM directly, no significant difference was observed (statistic not shown). Therefore, we then calculated the bacterial killing activity based on a protocol and formula by D. Ermert et al. [41] under MET‐inducing conditions again comparing m‐CSF to gm‐CSF hMDM. Interestingly, if we applied this calculation gm‐CSF hMDM showed significantly higher bactericidal capacity toward *E. coli* compared with m‐CSF hMDM (Figure 4D, red and green bars in Figure 4B,C to indicate the datasets used to calculate killing capacity). Since other bactericidal proteins such as histones and MPO could also be antibacterial, when bacteria are bound to ETs (Figure 4D, % killing; m‐CSF = 27.57% vs. gm‐CSF = 51.48% (mean)), we currently do not ascribe all the killing abilities of gm‐CSF METs solely to MMP12. Taken together, these data show that although m‐CSF‐differentiated macrophages cannot express MMP12 on day 3, this loss does not have a negative impact on their overall METosis capacity, but is instead associated with a significant reduction in the bactericidal capacity of these METs.

### 2.5 Calcium Induced MET Release Requires Enzyme Activity of Phospholipase C, PAD2, Mitochondrial ROS, and Plasma Membrane Rupture

We were now interested in obtaining some insights into how MET release is regulated in macrophages stimulated via extracellular Ca^2+^ and LPS.

**FIGURE 5:**
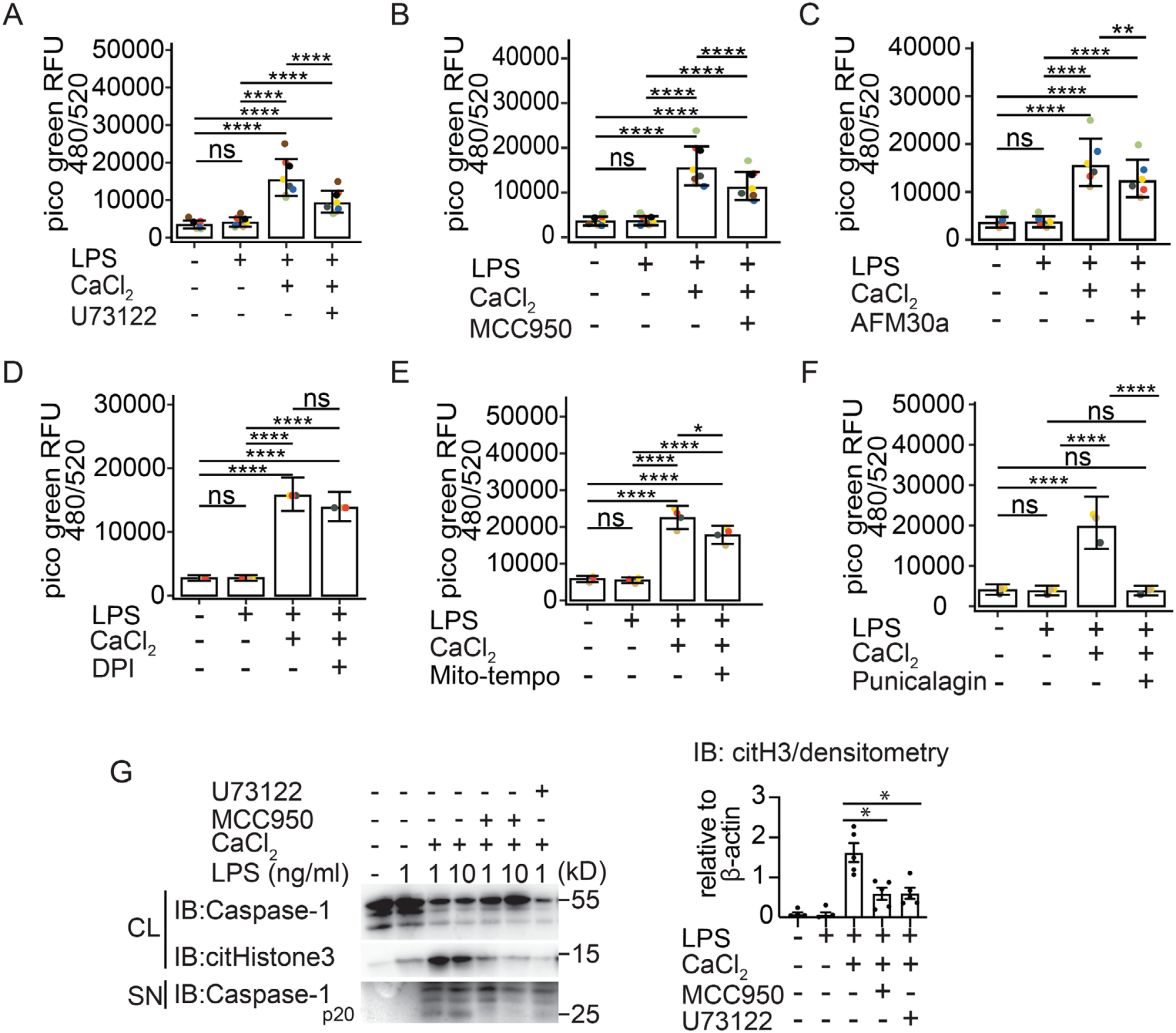
Phospholipase C and PAD2 activation together with plasma membrane rupture induces MET release: Quantification of MET release from gm‐CSF‐hMDM left untreated or primed for 2 h with LPS (1 ng/ml) or LPS primed and pretreated for 30 min with (A) U73122 (10 µM) or (B) MCC950 (2 µM) or (C) AFM30a (100 nM) and stimulated for 3 h with extracellular Ca^2+^ (1 mM) or (D) pretreated for 30 min with DPI (1 µM) or (E) pretreated for 30 min with mitoTEMPO (500 µM) and stimulated for 3 h with extracellular Ca^2+^ (1 mM) or (F) pretreated for 30 min punicalagin (25 µM) and stimulated for 3 h with extracellular Ca^2+^ (1 mM). (G) Immunoblot and quantification by band densitometry for caspase‐1 and citrullinated H3 from cell lysates and supernatants of gm‐CSF hMDM left untreated or primed for 2 h with LPS and activated for 3 h with extracellular Ca^2+^ (1 mM) in presence of inhibitors as indicated. ß‐actin serves as a loading control. Data information: Each dot represents an individual biological replicate. Data are representative of eight independent experiments (A) and (B) with technical triplicates, data are representative of six independent experiments with technical triplicates (C), data are representative of four independent experiments with technical duplicates (D), data are representative of three independent experiments with technical quadruplicates (E), data are representative of four independent experiments with technical triplicates (F), data are representative for one out of five independent experiments (G). Data are presented as the mean and 95% confidence limits. ns, not significant, ‘****’ *p* < 0.0001, ‘***’ *p* < 0.001, ‘**’ *p* < 0.01, ‘*’ *p* < 0.05. (Generalized linear mixed models for Gamma‐family with log‐link function and Tukey‐adjusted post hoc test [A–F]).

Since extracellular calcium activates macrophages via the calcium‐sensing receptor in a process dependent on phospholipase C activity [27], we pretreated primed gm‐CSF hMDM with the PLC inhibitor U73122. As expected, we observed a significant reduction in MET formation by blocking phospholipase C activity (Figure 5A). A similar effect of U73122 inhibition on the extracellular DNA release was observed in calcium‐activated m‐CSF hMDM (data not shown). Because extracellular Ca^2+^ also activates the NLRP3 inflammasome [27, 28, 29], we examined the effect of MCC950, which is a selective NLRP3 inflammasome inhibitor. Unexpectedly, NLRP3 inflammasome inhibition with MCC950 also significantly inhibited MET release (Figure 5B). Since we observed histone citrullination in macrophages stimulated with extracellular Ca^2+^ (Figure 1A,B) and PAD2 is the most strongly expressed PAD enzyme among the five PAD isoenzymes (PAD1‐4 and PAD6) in macrophages [31, 33] and because PAD enzymes are implicated in NET release in neutrophils [8, 34], we subsequently blocked PAD2 activation with a selective PAD inhibitor, AFM 30a. This also led to a significant reduction of MET release (Figure 5C) and histone citrullination (Figure S4A). Additionally, the impact of Cl‐amidine, a pan‐PAD inhibitor, on MET release was evaluated. Although there was a trend toward a reduction in MET release with Cl‐amidine, no significant inhibition was observed with extracellular Ca^2^⁺, with or without LPS (data not shown). We believe that the greater potency of AFM30a compared to Cl‐amidine in macrophages [33] may explain this difference.

We were then interested in how MET release is regulated further downstream in human MDM. Since we had observed a significant reduction in MET release with the NLRP3 inhibitor MCC950, we speculated, that activation of caspases could influence MET release in macrophages. However, using the pan‐caspase inhibitor z‐VAD‐FMK, we found that MET release was independent of caspase activation (Figure S4B). We therefore speculated that METs could be released via necroptosis, a cell death pathway that is dependent on RIP1 kinase activity [42]. Therefore, we tested the RIP1 kinase inhibitor, necrostatin, which also did not inhibit MET release (Figure S4B).

Since generation of ROS [6, 43] is required for NADPH‐dependent NETosis we next sought to investigate the requirements for ROS in our model. Inhibition of total ROS production by DPI treatment had no effect on MET release (Figure 5D) however inhibition of mitochondrial ROS production using a specific scavenger of mitochondrial superoxide (mitoTEMPO) attenuated MET release (Figure 5E).

We were now interested in finding out how the DNA is released from macrophages. GSDMD is activated downstream of NLRP3 and in LPS primed and extracellular Ca^2+^ activated hMDM (Figure S1A). It is further established that cleaved GSDMD induces small membrane pores of ∼18 nm in size [44] that enable IL‐1ß release [16, 17]. However, it is unknown, if GSDMD pores may also enable macrophages to release METs. We therefore tested the GSDMD inhibitor LDC7559, which blocks inflammasome‐mediated pore formation. Interestingly inhibition of GSDMD function did not impair MET release (Figure S4C). We, therefore, speculated that MET release may depend more on plasma membrane permeabilization and examined the effect of punicalagin on MET release, a polyphenolic compound that aids in lipid stabilization of the plasma membrane and consequently inhibition of permeability [45]. Interestingly, we observed a significant decrease in MET release in punicalagin‐treated and extracellular Ca^2+^ activated m‐CSF‐hMDM (Figure 5F; Video S3). A similar level of inhibition of extracellular DNA release was observed in calcium‐activated gm‐CSF hMDM in the presence of punicalagin (data not shown). Furthermore, the membrane stabilising effects of punicalagin blocked IL‐1ß release from these macrophages (Figure S4D).

Having established, that MET release from extracellular Ca^2+^ activated hMDM depends on PAD2 activation (Figure 5C) and maybe to some extent on mitochondrial ROS (Figure 5E), we were next interested in gaining some mechanistic insights regarding the rather unexpected caspase‐independent role of NLRP3 in the process of MET release. We therefore tested, if NLRP3 inflammasome inhibition with MCC950 had an effect on histone citrullination. Using a monoclonal antibody to detect histone H3 citrullination at positions R2, R8, and R17 we could indeed detect a reduction of histone H3 citrullination in the cell lysates of gm‐CSF differentiated macrophages treated with MCC950 compared with macrophages activated with LPS and extracellular Ca^2+^ (Figure 5G). Consistent with the strong effect of the PLC inhibitor U73122 on MET release (Figure 5A), we also observed a strong reduction of histone citrullination with this inhibitor (Figure 5G).

**FIGURE 6:**
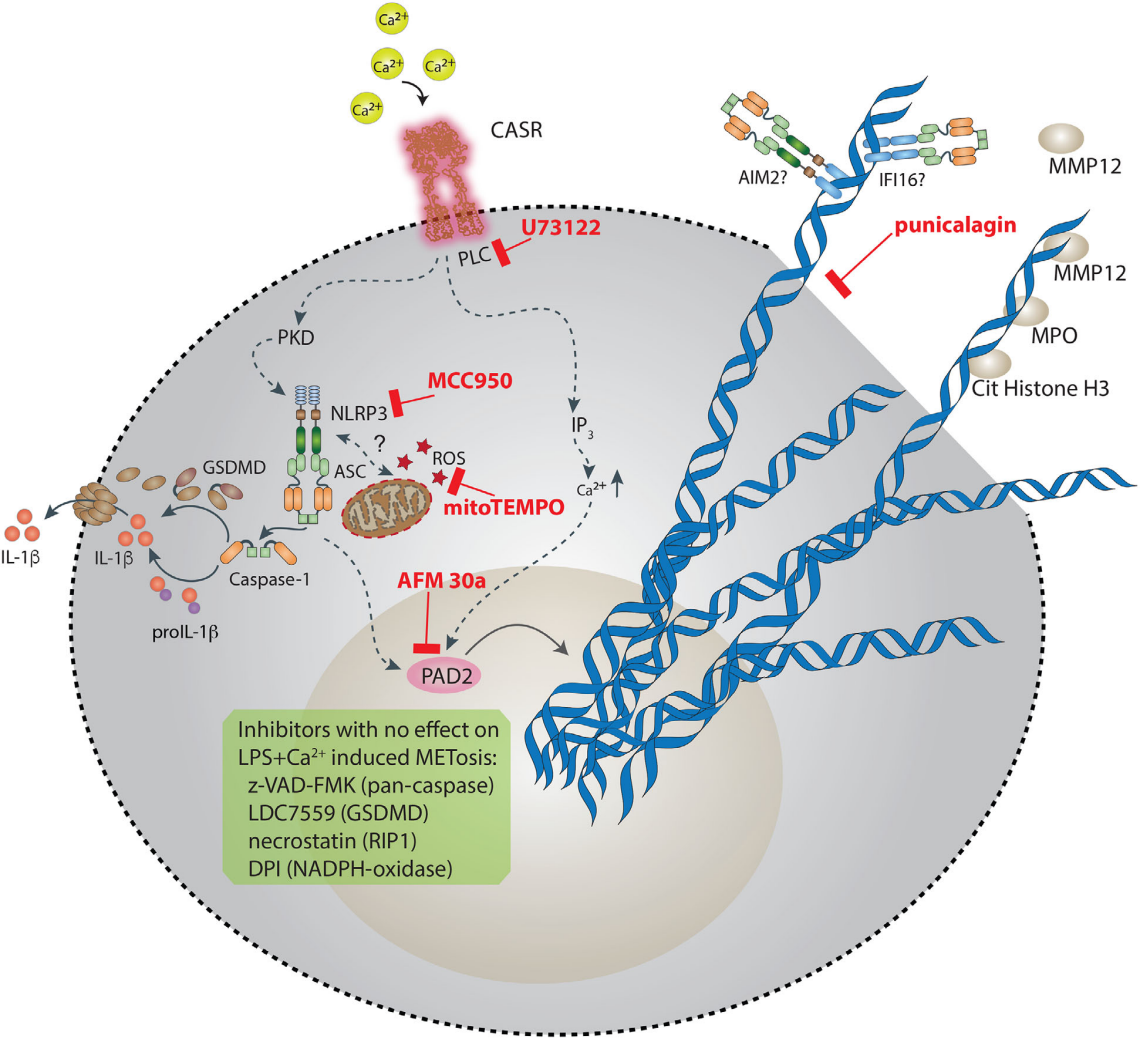
Proposed model of extracellular Ca^2+^ induced MET release: Our results support a model in which human monocyte‐derived macrophages release METs in response to extracellular calcium, a process that requires PLC, PAD2 activity, activity of the NLRP3 inflammasome, ROS and plasma membrane rupture. In LPS‐primed macrophages, extracellular calcium‐induced activation of the calcium‐sensing receptor triggers NLRP3 inflammasome assembly secondary to PLC activation and in the later stages GSDMD cleavage and proinflammatory IL‐1b release. Simultaneously, a PLC‐dependent increase in intracellular calcium may activate PAD2, which catalyzes histone citrullination and promotes MET formation. MET release is promoted by the release of mito‐ROS. NLRP3 seems to modulate histone citrullination through an unknown mechanism. Decondensed chromatin consists of citrullinated histone H3, MPO, ASC specks, and MMP12, and is released into the extracellular space by plasma membrane rupture.

This data together suggests a model of extracellular Ca^2+^ induced MET release, in which the extrusion of DNA depends on phospholipase C, the NLRP3 inflammasome, mito‐ROS, and PAD2 activity (Figure 6). Extracellular Ca^2+^ induced NLRP3 inflammasome activation also depends on phospholipase C activity and NLRP3 activation contributes to the process of MET formation most likely by a so far unrecognized effect of NLRP3 activation on histone citrullination. Moreover, our data suggest that MET release is dependent on plasma membrane permeabilization but not on GSDMD pore formation.

## 3 Discussion

Perturbations in calcium homeostasis are associated with many human pathologies [46, 47, 48] and thus extracellular and intracellular calcium levels are tightly regulated [49, 50]. Our data show for the first time, that elevated extracellular calcium triggers human macrophages to release DNA in a process similar to NETs (of note, the mean ionized calcium concentration in the human plasma is 1.2 mM [50]).

Importantly, calcium concentrations in extracellular fluids are particularly high at sites of injury and infection [51] and chronic inflammatory conditions are associated with deposition of calcium in tissues [52]. Calcium accumulated in atherosclerotic plaques [53], in granulomatous tissues [54] or in calcium pyrophosphate arthritis [55] may provide the reservoirs of calcium ions to trigger macrophages to release METs. Moreover, there is a link between inflammasome activation and extracellular calcium, since calcium‐induced IL‐1ß secretion is linked to the calcium‐sensing receptor in a process that requires NLRP3 activation downstream of phospholipase C activation [27].

When comparing the requirements for the release of extracellular DNA from macrophages with those from neutrophils, some differences are apparent from our study. Firstly, macrophages do not make METs in response to a TLR4 signal alone (Figure 1A,B; Figures S1B, 3C), whereas neutrophils do [34]. Secondly, even unprimed macrophages make METs in response to a subtle rise in extracellular calcium (Figure S1D), whereas it is currently unknown if elevated levels of extracellular calcium alone also trigger NETs in neutrophils [56]. Thirdly, following inflammasome activation IL‐1ß is released via gasdermin D pores [16, 17] and these pores have been proposed to be critical also for NET release [9]. However, this concept has recently been challenged [11]. We observed, that inflammasome activation occurs in parallel with extracellular calcium‐induced MET release, a finding, that is supported by the cleavage of GSDMD (Figure S1A), IL‐1ß secretion (Figure 1C), and identification of ASC specks (Figure 1A). Since both pathways, inflammasome activation and MET release converge under our assay conditions, we were interested to explore the requirement for GSDMD in the process of ETosis in human macrophages. In line with data by Stojkov et al. [11] using murine neutrophils we too did not find a requirement for GSDMD pores for human METs when employing the GSDMD inhibitor LDC7559 (Figure S4C). Interestingly, interfering with membrane permeabilization using punicalagin completely blocks extracellular DNA release from human macrophages (Figure 5F, Video S3).

Surprisingly, we found that inhibition of the NLRP3 inflammasome with MCC950 resulted in a decrease in histone citrullination (Figure 5G) and also significantly interfered with the amount of extracellular DNA released from human macrophages stimulated with LPS and extracellular Ca^2+^ (Figure 5G,B). Since this MCC950‐mediated reduction of DNA release was independent of caspase activation (Figure S4B) and GSDMD (Figure S4C), we currently assume, that either a previously unrecognized caspase‐independent link between NLRP3 activation and PAD activation exists or an off‐target effect of the inhibitor. Since macrophage cell lines such as THP‐1 cells may not always reflect the biological properties of human primary cells [57, 58, 59], we decided to work with primary human macrophages throughout this study; however, cell lines may help to investigate the possible connection between NLRP3 and PAD2 in the future.

The CASR is a G‐protein coupled receptor that signals via phospholipase C (PLC) to mobilize Ca^2+^ from intracellular stores. Although we currently do not know how exactly PLC activation is linked to MET release, some studies have suggested that PKC is activated further downstream of PLC [60]. Interestingly, some studies have also shown NADPH oxidase activation downstream of PKC [61]. In this study, extracellular calcium‐induced MET release was at least partially dependent on mitochondrial ROS production (Figure 5E). This shows, that ET release following activation of the CaSR via extracellular calcium is similar to ET release when using triggers such as calcium ionophores because stimulation of neutrophils with calcium‐ionophores does not require NADPH activity for NET release [5]. Interestingly, calcium ionophore‐stimulated human neutrophils were also reported to activate SK3 calcium channels in a process dependent on mitochondrial ROS but independent of NADPH [5, 6]. Since extracellular calcium‐induced MET release depends on mitochondrial ROS, we speculate, that next to extracellular calcium sensed by the CaSR maybe other calcium‐regulated channels exist, that may trigger MET release independent of the CaSR in human macrophages. This could explain, why PLC inhibition did not completely block MET release from human MDMs suggesting the existence of other pathways. Since MET formation may occur ROS‐dependent or ROS‐independent [23, 62], our data is in line with those reports demonstrating a role for mitochondrial ROS in MET formation [5, 6]. Further research is necessary to better understand how extracellular calcium‐induced mitochondrial ROS production and PLC activation are linked to MET formation in macrophages.

Currently, the role of PAD enzymes in the process of METosis has not been studied in detail. One study proposed, that MET release from human monocyte‐derived macrophages was independent of PAD2 expression [31], whereas another study found that MET release from M1 differentiated THP‐1 macrophages was PAD4 dependent [32]. Our data show prominent histone citrullination in extracellular Ca^2+^ activated macrophages (Figure 1A,B) and a PAD2 specific inhibitor, AFM 30a, significantly blocked MET release (Figure 5C) and histone citrullination (Figure S4A), suggesting that histone decondensation via citrullination promotes MET release.

For the first time, to our knowledge, we demonstrated that the macrophage polarization status could have a profound impact on the composition of METs. Although both polarization states enable macrophages to produce METs (Figure 3A,C) gm‐CSF differentiated hMDM may eject METs composed of more bactericidal proteins such as MPO (at day 6 differentiation, Figure 3B) and MMP12 (Figure 4A).

On the contrary, MMP12 was absent in day 3 differentiated m‐CSF‐hMDM (Figure 4A). This may have a profound impact on the ability of ETs to degrade virulence factors and/or to kill bacteria. Indeed, we found that METs released from gm‐CSF‐macrophages were much more potent in killing *E. coli* than those released from m‐CSF‐macrophages (Figure 4D).

Intriguingly, during a 6‐day differentiation, m‐CSF‐hMDM express less MPO (Figure 3B) but these macrophages were not impaired in their capacity of make METs (Figure 3C). This could be due to the presence of residual MPO expression in the 6 day differentiated m‐CSF‐hMDM (Figure 3b). Interestingly, by testing an MPO inhibitor we observed a significant reduction of MET release in gm‐CSF differentiated hMDM (Figure 3D). This observation is consistent with the findings of Metzler et al. [63], showing that patients with MPO deficiency have a lower ability to form NETs and that the amount of NETs produced correlates with the degree of MPO deficiency. However, we cannot explain how MPO might be important in MET release.

Moreover, we observed that the inflammasome adaptor protein ASC assembles around DNA fibers (Figure 1A; Video S1) released from macrophages, which now adds inflammasome complexes to the list of ET‐associated proteins. Further research is necessary to identify the DNA sensor protein by which extracellular ASC aggregates on these DNA fibers, most likely IFI‐16 [64] or AIM2 [65].

Our study also raises the question of the biological role of extracellular calcium‐induced METosis in vivo. In this context, it is interesting to note, that monocytes exposed to extracellular calcium were shown to upregulate the chemokine receptor CCR2 enhancing their responsiveness to MCP‐1, which is in turn produced by macrophages themselves and other cells at the site of inflammation [66]. This suggests a triple role for elevated extracellular calcium as an ionic chemokinetic, MET inducer, and inflammasome trigger that can modulate the innate immune response of monocytes and macrophages. We speculate, that mononuclear cells entering a site with an elevated level of extracellular calcium first fight off infections by phagocytotic means. However, if local calcium hemostasis is not restored within some time, macrophages could then engage in the fight by also extruding extracellular DNA traps and, at the same time release the alarm signals (via IL‐1 family members) to call for further help by IL‐1 responsive cells to the site of danger.

We believe that the identification of a reproducible condition for the generation of METs could help to precisely investigate the exact conditions for the release of extracellular DNA from macrophages and thus benefit in the search for potential therapeutics that could be important for the treatment of cardiovascular diseases, autoimmune diseases or infections. Further research on human macrophages is needed to decipher the exact signaling events that control METosis.

### 3.1 Materials and Methods

#### 3.1.1 Reagents and Antibodies

Ultrapure LPS (*E. coli* strain O111:B4) was from Invivogen (France) and calcium chloride solution and cytochalasin D was from Sigma‐Aldrich (Darmstadt, Germany). Quant‐iT PicoGreen was from Thermo Scientific (Darmstadt, Germany). Following primary antibodies were purchased from: anti‐histone H3 (ab5103), multiclonal anti‐histone H3 (ab281584), anti‐MMP12 (ab137443), anti‐MPO (EPR4793, ab 134132), all Abcam (Netherlands); anti‐caspase‐1 (2225) Cell Signaling; anti‐histone H3 (07‐690) Merck Millipore; anti‐PYCARD (1C3D7) and anti‐LAMP‐4/CD68 monoclonal antibody (KP1), Thermo Fischer; (Bio‐Techne, Wiesbaden, Germany); ß‐actin C4 (sc 47778), Santa Cruz. The following secondary antibodies were from Thermo Scientific: alexa fluor goat anti‐rabbit IgG 488 and alexa fluor donkey anti‐mouse IgG 647. Nuc blue live‐ready probes (Hoechst 33342), Sytox Green, and DRAQ5 were from Thermo Scientific. Antibodies CD86 (374207), CD80 (305217), CD40 (334305), CD163 (333625), CD206 (321131) for FACS assay were all from Biolegend. The ELISA kit for human IL‐1ß was from R&D Systems. Human CD14 microbeads and m‐CSF were from Miltenyi Biotec (Bergisch Gladbach, Germany). Recombinant human gm‐CSF was from Immunotools (Germany), CellGenix GMP DC medium was from CellGenix (Freiburg, Germany) and micrococcal nuclease was from Worthington Biochemical Corp (Cellsystems, Troisdorf, Germany). BD Cytofix/Cytoperm fixation/permeabilization kit was from BD Biosciences.

#### 3.1.2 Inhibitors

Punicalagin, diphenyleneidonium chloride, febuxostat, Cl‐amidine and mitoTEMPO were from Sigma‐Aldrich, MCC950, necrostatin‐1s and z‐VAD‐FMK was from Invivogen, U73122, MPO inhibitor (MPO‐IN‐28), and LDC7559 was from Medchem express (Hycultec, Beutelsbach, Germany). AFM 30a was from P. Thompson (University of Massachusetts Medical School, Worcester, MA).

#### 3.1.3 Differentiation and Stimulation of Human Primary Macrophages

Buffy coats from healthy donors were obtained at the University Medicine Greifswald (ethical approval: BB014‐14). PBMCs were isolated from buffy coats using bicoll density gradient centrifugation. PBMCs were incubated with CD14 magnetic microbeads at 4°C according to the manufacturer's instructions. Labeled CD14^+^ monocytes were isolated using MACS column placed on magnetic MACS separator. CD14^+^ monocytes were seeded at 1 × 10^6^ cells/mL in 12‐well plates in CellGenix GMP DC medium containing 500 U/mL rhGM‐CSF or m‐CSF to generate monocyte derived‐macrophages for 3, 5, or 6 days. Differentiated hMDM were primed with LPS (1 ng/mL, unless otherwise indicated) in RPMI media for 2 h and activated with extracellular Ca^2+^ (1 mM, unless otherwise indicated) for indicated time intervals. Inhibitors were added after LPS priming for 30 min at the following concentration: MCC950 (2 µM), LDC7559 (2 µM), febuxostat (200 µM), DPI (1cµM), mitoTEMPO (500 µM), AFM 30a (100 nM), punicalagin (25 µM), U73122 (10 µM), z‐VAD‐FMK (20 µM), necrostatin‐1s (10 µM), Cl‐amidine (200 µM), and MPO inhibitor (5 µM). Flow cytometry analysis to confirm the phenotype of gm‐CSF and m‐CSF macrophages was performed according to the manufacturer's instructions. gm‐CSF‐ and m‐CSF‐differentiated macrophages were fixed on ice with 250 µL Cytofix (BD Biosciences) for 20 min. After washing with the permeabilization medium Cytoperm (BD Bioscience), fixed samples were stained with lineage‐specific antibodies (CD40‐FITC; CD80‐PE/Cy7; CD86‐APC; CD163‐PerCP; CD206‐AF700; all 1:100) for 1 h on ice. Cells were washed again with 1 mL permeabilization medium at 600 g for 8 min and flow cytometric analysis was performed on a BD FACS Aria III (BD Biosciences).

#### 3.1.4 Immunofluorescence

Macrophages seeded onto 10 mm glass coverslips (Carl Roth, Germany) were left untreated or primed with LPS or primed with LPS and stimulated with extracellular Ca^2+^. Cells were then fixed with 4% paraformaldehyde at RT, 20 min [35] and blocked with blocking buffer (1% BSA, 0.3 M glycine, 0.1% Tween‐20, 1% goat or donkey sera as needed in PBS), 1 h, RT and incubated in blocking buffer with the primary antibodies anti‐PYCARD (1C3D7), anti‐histone3 (ab5103), anti‐MPO (ab134132), anti‐MMP12 (ab137443), LAMP4/anti‐CD68 (KP1) at 1:100, overnight, 4°C. On the next day, after 3 washes in PBS, secondary antibodies goat anti‐rabbit IgG 488, donkey anti‐mouse IgG‐647 were used at 1:100 in blocking buffer, 1 h, RT and DNA was counterstained with Hoechst 33342 (Invitrogen). Coverslips were mounted with fluorescence mounting media (Dako) and analyzed on a Leica TCS‐SP5 confocal microscope (Leica Microsystems). Images were analyzed using ImageJ (US National Institutes of Health).

#### 3.1.5 MET Quantification

Differentiated macrophages seeded at 1 × 10^6^ cells/mL were left untreated, primed with LPS or primed with LPS and activated with extracellular Ca^2+^ for indicated time intervals. METs were quantified as described by Fuchs et al. [35]. Briefly, after macrophage stimulation, the cell culture plate was spun at 300 g, 5 min and supernatant were harvested for cytokine measurements. Extracellular DNA was digested with 500 mU/mL micrococcal nuclease in HBSS with calcium for 20 min at 37°C. Nuclease activity was stopped with 5 mM EDTA and the plate was spun at 1500 g, 5 min, 4°C. MNase digested supernatant was transferred into a new plate and DNA was quantified using PicoGreen according to manufacturer's instructions.

#### 3.1.6 Live Cell Imaging

CD14+ monocytes were seeded onto 35 mm‐glass bottom culture dishes (Mattek Corp, Germany) and differentiated into macrophages in CellGenix GMP DC medium containing 500 U/mL rhGM‐CSF for 3 days. Differentiated macrophages were incubated in 4 mL DMEM media without phenol red containing 20 mM HEPES, 2 µM DRAQ5, and 0.5 µM Sytox Green at 37°C, 20 mins. Cells were then primed with LPS (1 ng/mL) or pretreated for 30 min with punicalagin (25 µM) and stimulated with extracellular Ca^2+^ (1 mM). Imaging was performed on a Leica TCS‐SP5 confocal microscope (Leica Microsystems) in the temperature‐controlled chamber at 37°C for over 3 h. Images were acquired at 488 laser power (4%) and 633 laser power (9%), every 3 min.

#### 3.1.7 Western Blot

gm‐CSF or m‐CSF hMDM were seeded at 1 × 10^6^ cell/mL in 12–well. hMDM were left unstimulated or primed with LPS and stimulated for 3 h with extracellular Ca^2+^ (1 mM). Cell lysates were prepared in RIPA buffer, resolved by 12% SDS‐PAGE electrophoresis, and transferred to PVDF. Following primary antibodies were used: anti‐histone H3 (ab5103), anti‐histone H3 (ab281584), anti‐histone H3 (07‐690), anti‐MPO (ab134132), anti‐MMP12 (AF917), anti‐GSDMD (96458S), anti‐caspase‐1 (2225, cell signaling), and ß‐actin (sc 47778) in 1:1000 in 5% skim milk +TBST, overnight, 4°C. On the next day, after five washes with TBST, HRP‐conjugated secondary antibodies were added at 1:5000, 1 h, RT and analyzed using the chemiluminescent detection method.

#### 3.1.8 Bacterial Killing Assay

CD14+ monocytes were seeded at 1 × 10^6^ cell/mL in 12‐well and differentiated into macrophages in the presence of 500 U/mL rhGM‐CSF or m‐CSF for 3 days. Differentiated macrophages were either left untreated or primed for 2 h with LPS 1 ng/mL and activated for 3 h with extracellular Ca^2+^ (1 mM) in duplicates or triplicates. After stimulation, supernatants were harvested without disturbing METs and 450 µL fresh RPMI (without phenol red) with 10 mM HEPES was added. To inhibit phagocytosis, macrophages were incubated with cytochalasin D (10 µg/mL), 15 min, 37°C, 5% CO_2_. Prior to adding *E. coli*, LPS and calcium‐activated samples were incubated with MNase 100 U/mL for MET digestion. Thereafter, 50 µl of *E. coli* suspension at MOI of 1 was added to each condition in the final assay volume of 500 µL and was incubated at 37°C, 1 h. After incubation, wells were scraped to remove any attached METs and *E. coli*. The suspension from each condition was diluted 10^−3^ in LB media and 100 µL of diluted supernatant was plated on LB‐containing plates and incubated at 37°C, 16–20 h. On the next day plates with colony forming units (CFU) counts were manually counted. Percent survival and killing was calculated as described by Ermert et al. [41] using the following formulas:

1. % survival = (dilution factor × CFU _with macrophages_ ÷ dilution factor × CFU *_E. coli_*) × 100
2. Mean values from duplicates or triplicates were used to determine average CFU
3. % MET killing = (dilution factor × CFU _with MNase_ − dilution factor × CFU _without MNase_ ÷ dilution factor × CFU _with MNase_) × 100

#### 3.1.9 *E. coli* Culture

Glycerol stock of *E. coli* XL1‐Blue (Agilent) was thawed on ice and 10 µL bacterial suspension was added to LB media. Bacterial culture was grown at 37°C, 4–5 h until O.D _600_ of 0.3–0.4 was reached.

#### 3.1.10 Statistical Analyses

Prism software (GraphPad Software, Inc) and R version 4.2.2 with packages ‐lmerTest‐, ‐car‐, ‐emmeans‐, and ‐ggplot2‐ were used for all statistical analyses. If not otherwise given mean values with 95% confidence limits are presented in the barplots with donor‐specific means over the replicates given as dots (each color represents one individual blood donor but only for the given experiment). We used generalized linear mixed effects models (GLM for Gamma‐family with log‐link function) to account for the clustering (replicates per donor) and right‐skewness in the data. Testing different inhibitors, the following treatment groups were compared in the GLMs in a 1‐way ANOVA‐like analysis: none/LPS/LPS+CaCl_2_/LPS+CaCl_2_+Inhibitor. Tukey adjusted post hoc tests were used to detect significant differences between treatment groups. In the experiments using gmCSF and mCSF generated macrophages on days 3 and 6, the main effects of treatment groups (none/LPS/LPS+CaCl_2_/LPS+CaCl_2_+Inhibitor), macrophage stimulus type (gmCSF / mCSF) and day (3/6) including their interactions were used in the GLMs to define three‐way ANOVA‐like treatment groups. We used a backward‐selection procedure to keep only significant interactions in a parsimonious model. Again, Tukey‐adjusted post hoc tests were used to compare the different treatment groups.

## Author Contributions

**Neha Mishra**: Conceptualization; data curation; formal analysis; investigation; methodology; writing–original draft; writing–review; **Magdalena Mohs**: data curation and formal analysis; **Nico Wittman**: FACS acquisition and formal analysis; **Stefan Gross**: Statistical analysis and data presentation; **Paul R. Thompson**: Resources; **Lukas Bossaller**: Resources, data curation; formal analysis; supervision; funding acquisition; investigation; methodology; writing–original draft; writing–review and editing; project administration.

## Ethics Statement

The study was conducted by the Declaration of Helsinki and approved by the Ethics Committee of the University Medicine Greifswald (protocol code BB 014/14, February 11, 2014). No animal experiments were performed in this study.

## Conflicts of Interest

P.R.T. is a consultant for Celgene and Disarm Therapeutics, founded Padlock Therapeutics, and has received fees from Bristol Myers Squibb. The remaining authors declare no conflicts of interest.

### Peer Review

The peer review history for this article is available at https://publons.com/publon/10.1002/eji.202350942

## Supporting information



Supporting Information

Supporting Information

Supporting Information

Supporting Information

## Data Availability

The data that support the findings of this study are available on request from the first and/or the corresponding author.
