# Supplementary material for: PLC and PAD2 Regulate Extracellular Calcium‐Triggered Release of Macrophage Extracellular DNA Traps
Source: Eur J Immunol. 2025 Apr 1;55(4):e202350942. doi: 10.1002/eji.202350942 (PMC11962252; doi:10.1002/eji.202350942)
Supplement: Supplementary file 1 — Supporting Information [file EJI-55-e202350942-s002.pdf]

Supplementary Figures

PLC and PAD2 regulate extracellular calcium-triggered release of macrophage extracellular DNA traps

Neha Mishra, Magdalena Mohs, Nico Wittmann, Stefan Gross, Paul R. Thompson and Lukas Bossaller

Corresponding author: Lukas Bossaller, MD
Section of Rheumatology, Department of Medicine A, University Medicine Greifswald, Ferdinand-Sauerbruch-Strasse, 17475 Greifswald, Germany.
Phone: +49 3834 866660, Fax: +49 3834 86 6681
e-mail: lukas.bossaller@med.uni-greifswald.de

European Journal of Immunology: 10.1002/eji.202350942

Supplementary Figure 1

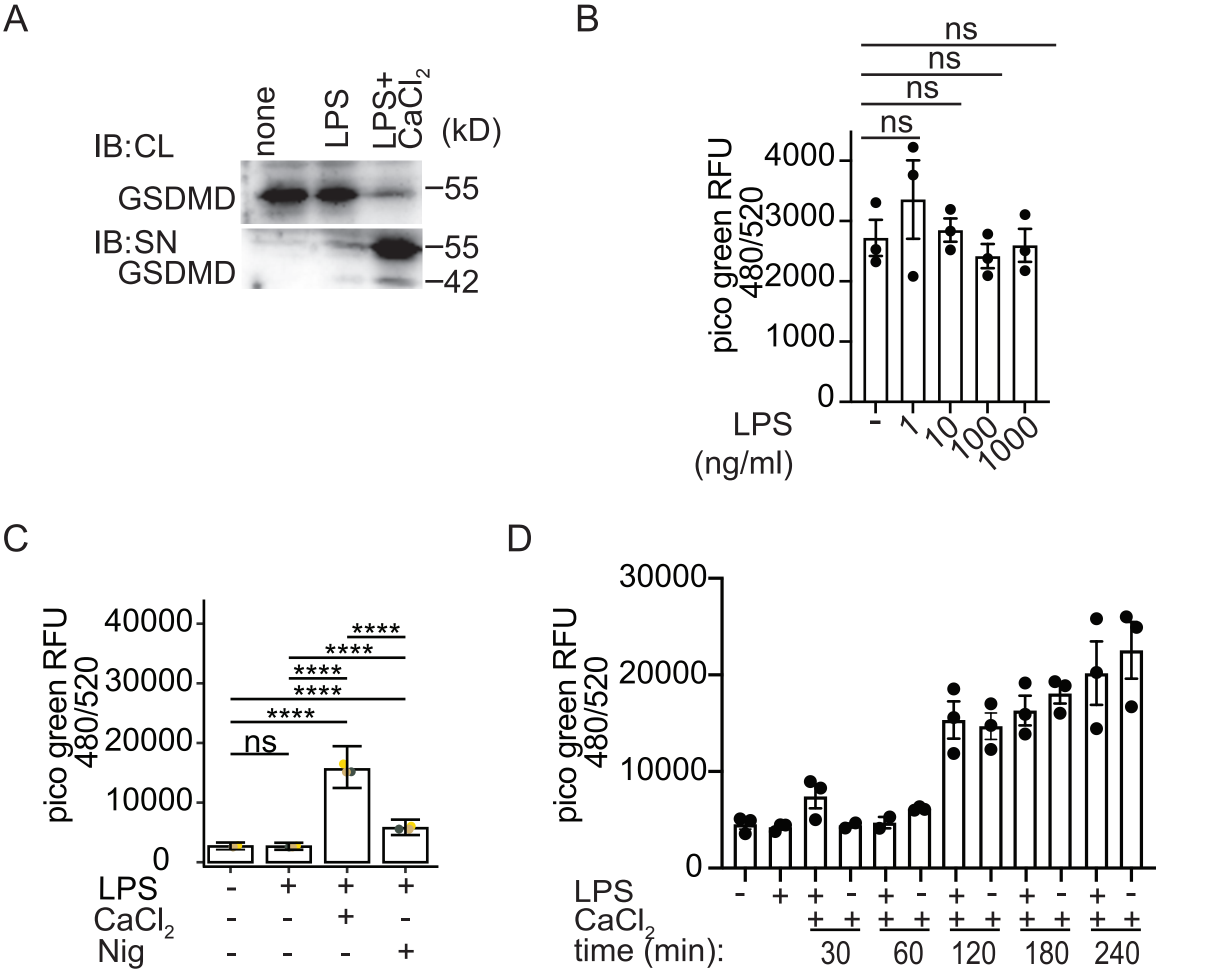

Supplementary Figure 1. Extracellular Ca^{2+} induces GSDMD cleavage and LPS alone does not induce MET release while extracellular Ca^{2+} alone does induce MET release from human MDM:

(a) Immunoblot for GSDMD from cell lysates and supernatants of gm-CSF hMDM left untreated or primed for 2 h with LPS (1 ng/ml) or primed with LPS (1 ng/ml) and activated for 3 h with extracellular Ca^{2+}(1 mM). (**b**) Quantification of MET release from gm-CSF-hMDM left untreated or primed for 6 h with LPS as indicated. **(c)** Quantification of MET release from gm-CSF-hMDM left untreated or primed for 2 h with LPS (1 ng/ml) or primed for 2 h with LPS (1 ng/ml) and stimulated for 3 h with nigericin (5 µM) or extracellular Ca^{2+} (1 mM). **(d)** Quantification of MET release from gm-CSF-hMDM left untreated or primed for 2 h with LPS (1 ng/ml) or primed for 2 h with LPS (1 ng/ml) and stimulated with extracellular Ca^{2+} (1 mM) or left unprimed and stimulated with extracellular Ca^{2+}(1mM) as indicated.

Data information: Each dot represents an individual biological replicate (B-D). Data from one experiment (**a**), data are representative of three independent experiments with technical triplicates (**b**), data are representative of three independent experiments with technical triplicates (**c**), data are representative of three or two independent experiments with technical duplicates (**d**). Data information: Data are presented as the mean and 95% confidence limits. ns, not significant, '****' $p<0.0001$, '***' $p<0.001$, '**' $p<0.01$, '*' $p<0.05$ (Generalized linear mixed models for Gamma-family with log-link function and Tukey-adjusted post-hoc test (C)).

Supplementary Figure 2

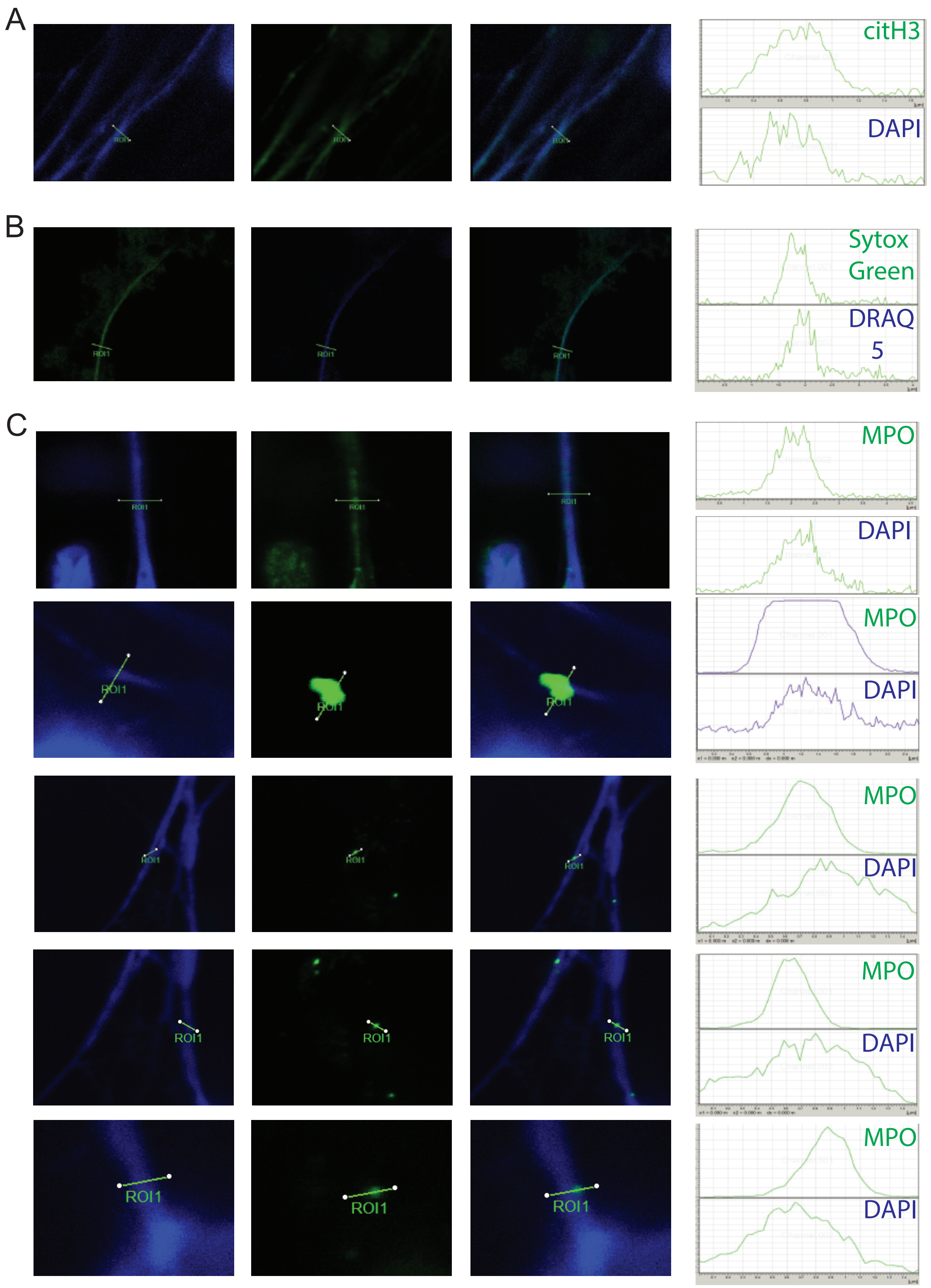

D

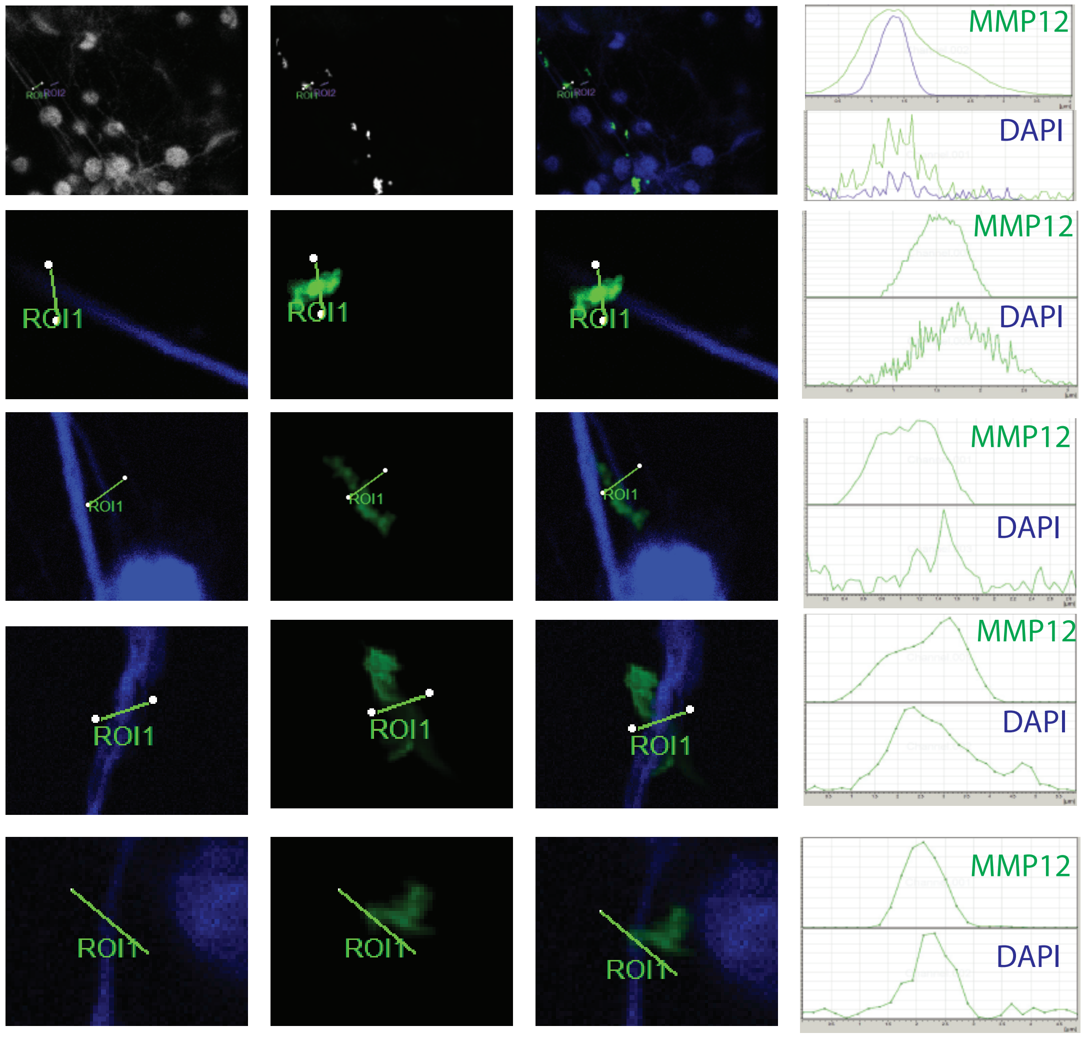

Supplementary Figure 2. Citrullinated Histone H3, MPO and MMP12 are associated with METs: Confocal microscopy with line analysis to show pixel intensity (y axis) along the indicated line (x axis) in each of the two fluorescence channels in LPS (1ng/ml) primed and extracellular Ca^{2+} (1mM) activated hMDM stained for (a) citrullinated H3 and DAPI or (b) Sytox Green and DRAQ5 or (c) MPO and DAPI or (d) MMP12 and DAPI.
Data information: Data are representative of one from three independent experiments (a), or three independent experiments (b) or five independent experiments (c) and (d).

Supplementary Figure 3

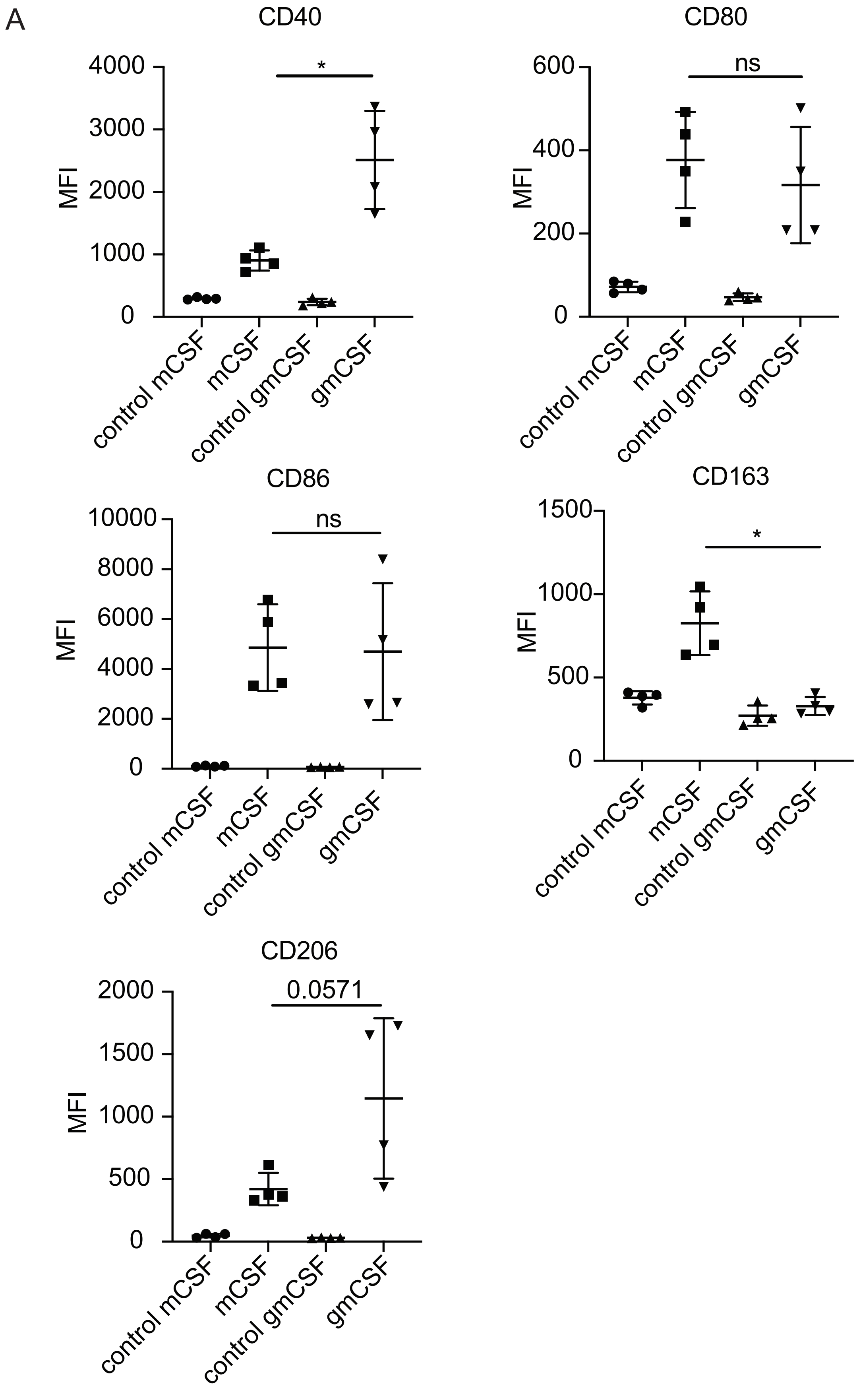

European Journal of Immunology: 10.1002/eji.202350942

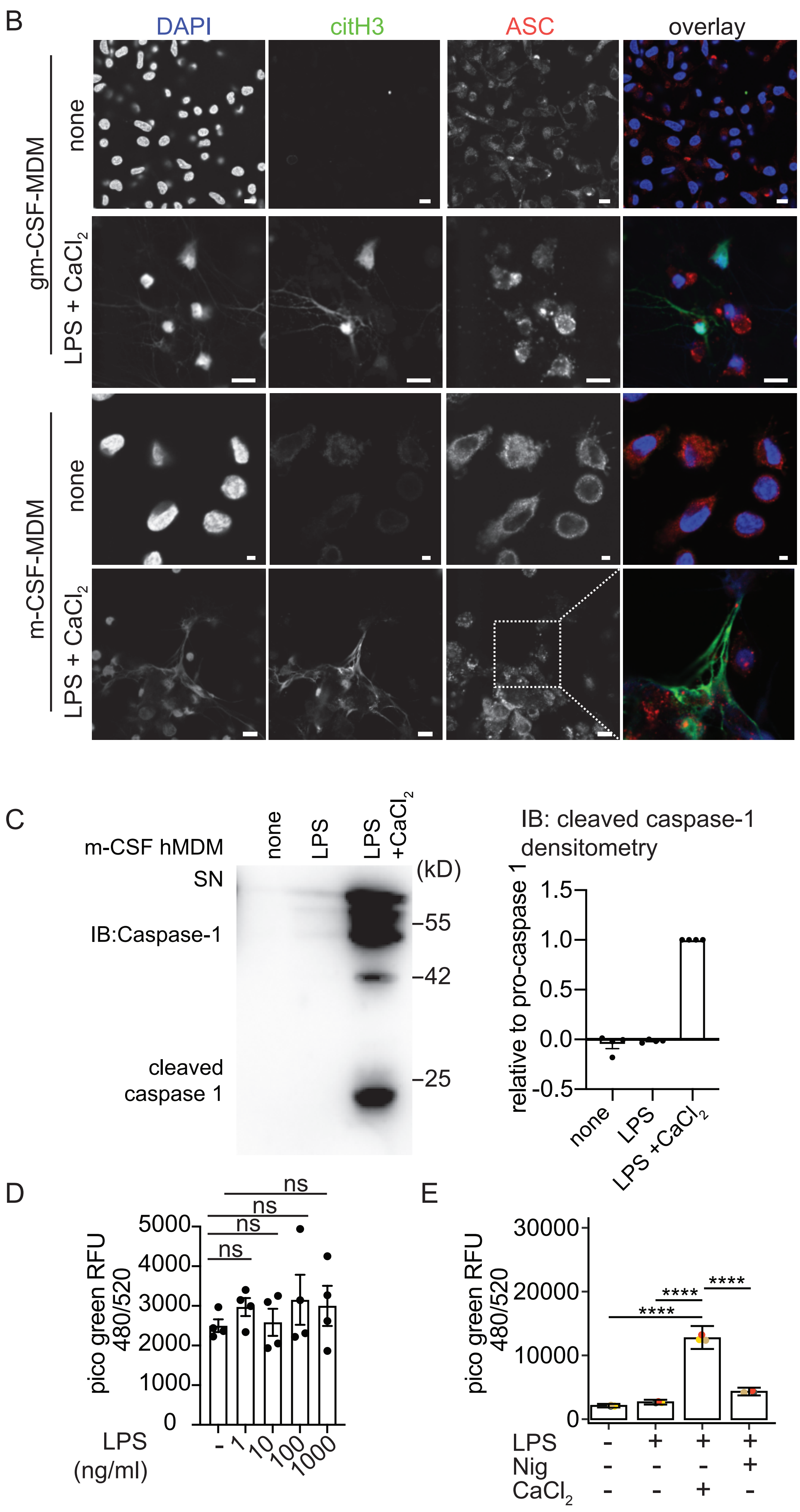
B
DAPI
citH3
ASC
overlay
gm-CSF-MDM
none
LPS + CaCl2
m-CSF-MDM
none
LPS + CaCl2
C
m-CSF hMDM
SN
IB:Caspase-1
cleaved caspase 1
none
LPS
LPS +CaCl2
(kD)
–55
–42
–25
IB: cleaved caspase-1 densitometry
relative to pro-caspase 1
1.5
1.0
0.5
0.0
-0.5
none
LPS
LPS +CaCl2
D
pico green RFU 480/520
5000
4000
3000
2000
1000
0
ns
ns
ns
ns
LPS (ng/ml)
-
1
10
100
1000
E
pico green RFU 480/520
30000
20000
10000
0
****
****
****
LPS
Nig
CaCl2

Supplementary Figure 3. LPS priming and extracellular Ca^{2+} induces inflammasome activation in gm-CSF and m-CSF-hMDM and LPS alone does not induce MET release from m-CSF-hMDM: (a) The mean fluorescence intensity of CD40, CD80, CD86, CD163 and CD206 surface markers expressed in gm-CSF and m-CSF differentiated hMDM. **(b)** Confocal microscopy of gm-CSF or m-CSF differentiated hMDM stained for citH3 (green), ASC (red), DNA (blue) left untreated or LPS primed and stimulated for 3 h with extracellular Ca^{2+} (1mM). **(c)** Immunoblot and quantification by band densitometry for caspase-1 from supernatants of m-CSF-hMDM left untreated or primed for 2 h with LPS (1ng/ml) or primed with LPS (1ng/ml) and activated for 3 h with extracellular Ca^{2+} (1mM). (**d**) Quantification of MET release from m-CSF-hMDM left untreated or primed for 6 h with LPS as indicated. (**e**) Quantification of MET release from m-CSF-hMDM left untreated or primed for 2 h with LPS (1 ng/ml) or primed for 2 h with LPS (1 ng/ml) and stimulated for 3 h with nigericin (5 µM) or extracellular Ca^{2+} (1 mM).

Data information: Each dot represents an individual biological replicate (A, C-D). Data are representative of four independent experiments (**a**). Data are from two or three independent experiments **(b),** data representative of one from four biological experiments **(c).** Data are representative of four independent experiment in triplicates (**d**) or three independent experiment in triplicates (**e**). Data information: Data are presented as the mean and 95% confidence limits. ns, not significant, '****' p<0.0001, '***' p<0.001,'**' p<0.01, '*' p<0.05 (One-way ANOVA, post hoc analysis). Scale bar all 10µM except for none in (**b**) 7.5µM.

Supplementary Figure 4

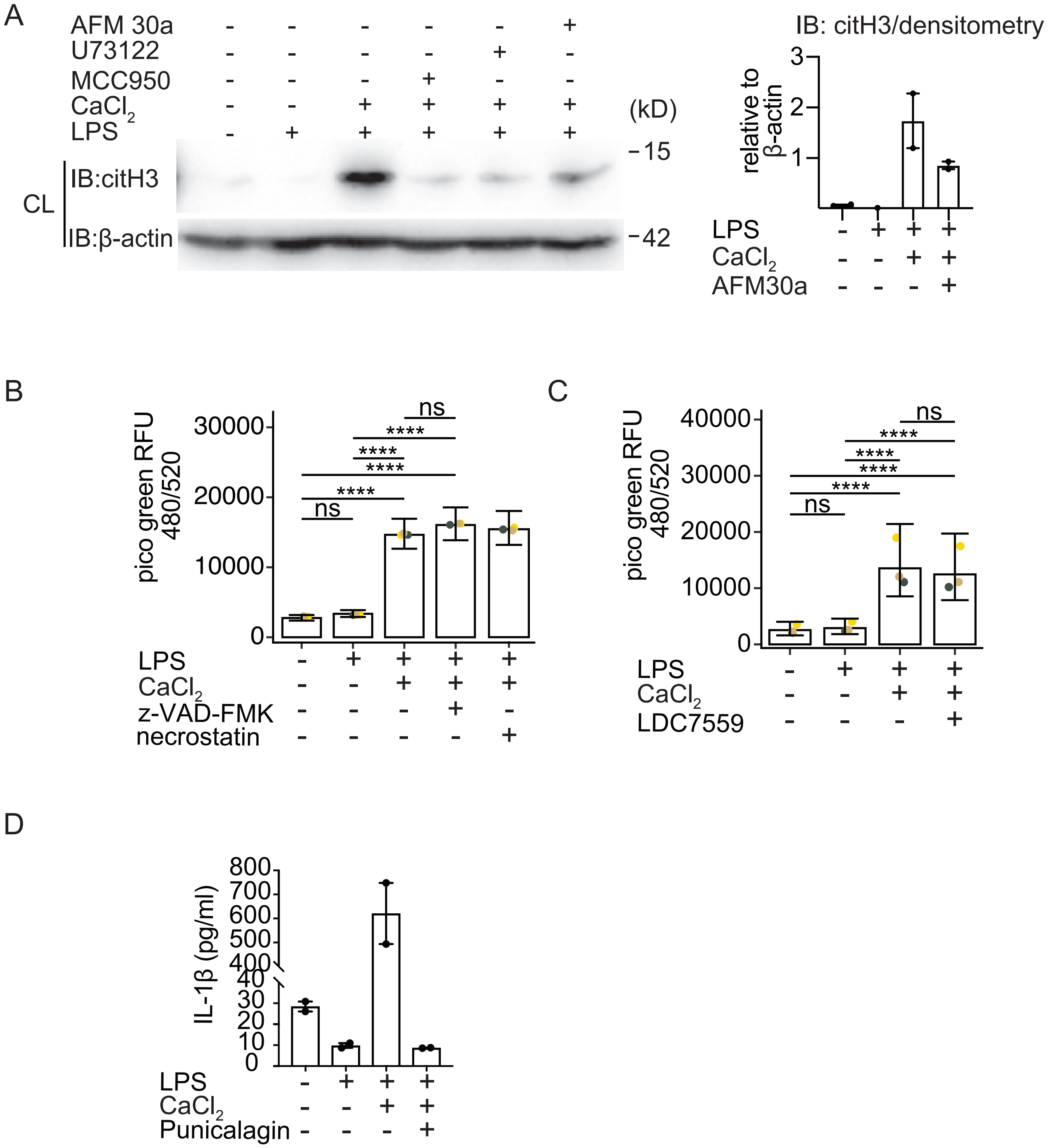

European Journal of Immunology: 10.1002/eji.202350942

Supplementary Figure 4. Caspases and RIP-1 kinase are not required for MET formation, however inhibition of plasma membrane permeabilization blocks IL-1b release from extracellular Ca^{2+} activated hMDM and PAD2 inhibition reduces histone H3 citrullination in LPS primed and extracellular Ca^{2+} activated gm-CSF-hMDM: (a) Immunoblot for citrullinated histone H3 from cell lysates of gm-CSF hMDM left untreated or primed for 2 h with LPS (1ng/ml) or primed with LPS (1ng/ml) and pretreated with MCC950 (2 µM) or U73122 (10 µM) or AFM 30a (100 nM) and activated for 3 h with extracellular Ca^{2+} (1mM). Beta-actin serves as a loading control. Data are representative of two biological experiments and quantification by band densitometry. (**b**) Quantification of MET release from gm-CSF-hMDM left untreated or primed for 2 h with LPS (1ng/ml) and pretreated for 30 min with z-VAD-FMK (20 µM) or necrostatin (10 µM) or **(c)** LDC7559 (2 µM) and stimulated for 1 h with extracellular Ca^{2+} (1 mM). **(d)** ELISA for IL-1ß of supernatants from inactivated hMDM (-) or primed for 2 h with LPS (1 ng/ml) or LPS primed and pretreated with punicalagin (25 µM) and stimulated for 3 h with extracellular Ca^{2+} (1 mM).
Data information: Each dot represents an individual biological replicate (B-D). Data are representative of three independent experiment in triplicates **(b)** or duplicates **(c)** or data representative of two independent experiments **(d)**. Data information: Data are presented as the mean and 95% confidence limits. ns, not significant, '****' $p<0.0001$, '***' $p<0.001$,'**' $p<0.01$, '*' $p<0.05$ (Generalized linear mixed models for Gamma-family with log-link function and Tukey-adjusted post-hoc test (B,C)).

Supplementary Video 1: ASC speck assembles around DNA: Z-stack confocal images of gm-CSF-hMDM stained for ASC (red), cit H3 (green) and DAPI (blue) primed for 2 h with LPS (1 ng/ml) and stimulated for 3 h with extracellular Ca^{2+}(1 mM).

Supplementary Video 2: Extracellular calcium induces MET release in gm-CSF-hMDM: Live cell confocal microscopy of hMDM stained with DRAQ5 (blue) and Sytox Green (green) primed with LPS (1 ng/ml) and stimulated with extracellular Ca^{2+} (1 mM). Images were taken every 3 mins for 3 h.

Supplementary Video 3: Plasma membrane stabilization inhibits MET release in LPS primed and extracellular Ca^{2+} activated gm-CSF-hMDM: Live cell confocal microscopy of hMDM stained with DRAQ5 (blue) and Sytox Green (green) primed with LPS (1 ng/ml) and pretreated with punicalagin (25 µM) and stimulated with extracellular Ca^{2+} (1 mM). Images were taken every 3 mins for 3 h.
